# Investigate the mechanisms of Chinese medicine *Fuzhengkangai* towards EGFR mutation-positive lung adenocarcinomas by network pharmacology

**DOI:** 10.1186/s12906-018-2347-x

**Published:** 2018-11-06

**Authors:** Zhitong Bing, Zhiyuan Cheng, Danfeng Shi, Xinkui Liu, Jinhui Tian, Xiaojun Yao, Jingyun Zhang, Yongfeng Wang, Kehu Yang

**Affiliations:** 1 Evidence Based Medicine Center, School of Basic Medical Science of Lanzhou University, Lanzhou, China; 2 Key Laboratory of Evidence Based Medicine and Knowledge Translation of Gansu Province, 199 West Donggang Road, Lanzhou, 730000 Gansu China; 3 Institute of Modern Physics of Chinese Academy of Sciences, Lanzhou, Gansu Province China; 4 Department of Chemistry, State Key Laboratory of Applied Organic Chemistry, Lanzhou University, Lanzhou, China; 5 Department of Clinical Chinese Pharmacy, School of Chinese Materia Medica, Beijing University of Chinese Medicine, Beijing, China; 6 Gansu University of Chinese Medicine, Lanzhou, China

**Keywords:** Systems pharmacology, Fuzhengkangai formula, Herbal medicines, Molecular docking

## Abstract

**Background:**

Chinese traditional herbal medicine Fuzhengkangai (FZKA) formulation combination with gefitinib can overcome drug resistance and improve the prognosis of lung adenocarcinoma patients. However, the pharmacological and molecular mechanisms underlying the active ingredients, potential targets, and overcome drug resistance of the drug are still unclear. Therefore, it is necessary to explore the molecular mechanism of FZKA.

**Methods:**

A systems pharmacology and bioinformatics-based approach was employed to investigate the molecular pathogenesis of EGFR-TKI resistance with clinically effective herb formula. The differential gene expressions between EGFR-TKI sensitive and resistance cell lines were calculated and used to find overlap from targets as core targets. The prognosis of core targets was validated from the cancer genome atlas (TCGA) database by Cox regression. Kyoto Encyclopedia of Genes and Genomes (KEGG) pathway enrichment is applied to analysis core targets for revealing mechanism in biology.

**Results:**

The results showed that 35 active compounds of FZKA can interact with eight core targets proteins (ADRB2, BCL2, CDKN1A, HTR2C, KCNMA1, PLA2G4A, PRKCA and LYZ). The risk score of them were associated with overall survival and relapse free time (HR = 6.604, 95% CI: 2.314–18.850; HR = 5.132, 95% CI: 1.531–17.220). The pathway enrichment suggested that they involved in EGFR-TKI resistance and non-small cell lung cancer pathways, which directly affect EGFR-TKI resistance. The molecular docking showed that licochalcone a and beta-sitosterol can closely bind two targets (BCL2 and PRKCA) that involved in EGFR-TKI resistance pathway.

**Conclusions:**

This study provided a workflow for understanding mechanism of CHM for against drug resistance.

**Electronic supplementary material:**

The online version of this article (10.1186/s12906-018-2347-x) contains supplementary material, which is available to authorized users.

## Background

Lung cancer is a leading cause of cancer mortality worldwide, more than 85% of which is non-small cell lung cancer (NSCLC). Lung adenocarcinoma is the major form of NSCLC, which represents about 50% of lung cancer [1]. Epidermal growth factor receptor (EGFR) mutation is a main contributing factor of lung adenocarcinoma (LUAD) in east Asian countries (about 60% of lung adenocarcinoma) [2]. In China, according to cancer statistics for 2015, lung cancer shows the highest morbidity and mortality [3].

The epidermal growth factor receptor tyrosine kinase inhibitors (EGFR-TKIs) such as gefitinib, erlotinib and afatinib, which targeted the EGFR pathway, showed potential in the treatment of patients with EGFR mutated NSCLC [4]. And the drugs have effects on LUAD patients with EGFR mutations including the deletion of exon 19 and L858R missense mutation of exon 21 [5]. Although they are effective for early treatment of LUAD, patients will soon have drug resistance in 4 to 12 months during therapy process [2]. Researchers have made great efforts to explore resistance mechanisms and they have discovered many mechanisms of EGFR-TKI resistance. The most frequently studied mechanism of acquired resistance is the T790 M point mutation in exon 2 of EGFR [6, 7]. Secondly, in histologic transformation, the small cell of LUAD histologic transformation and epithelial-mesenchymal transition (MET) activation were closely associated with the acquired EGFR-TKI resistance in patients with never smoked [8–11]. MET and HER2 amplification are also reported to associate with EGFR-TKI resistance [12–14]. BRAF secondary mutations have also been implicated to EGFR-TKI resistance [15]. In recent studies, PAK1 activation, upregulation of BCL2, elevation of CDKN1A (p21), overexpression of PHGDH and IGF1R related with acquire resistance [2, 16–19]. To sum up, so many mechanisms of drug resistance are very harmful to the target treatment of patients. How to overcome various anti-drug mechanisms is the focus of attention of many scientists.

Previous studies reported that Chinese Herbal Medicine (CHM) Fuzhengkangai (FZKA) formulation has a good performance in clinical cancer treatment [20, 21]. In recent year, Yang et al. reported that CHM of FZKA combine gefitinib had great effect to treat lung adenocarcinoma with EGFR mutation patients [22]. The study indicates that CHM combination of gefitinib can improve relapse free survival (RFS) significantly. However, the mechanism of CHM in LUAD remain unclear. With the deepening of network pharmacology research, an increasing number of the mechanism of CHM has been revealed [23–25]. Thus, this study employed network pharmacology, bioinformatics and molecular docking method to investigate the molecular mechanism of FZKA in against drug resistance.

## Methods

### Composite of Chinese herbs of FZKA

Previous publication has reported the composition of FZKA [22]. This prescription involved eleven herbs which contained *Atractylodes Macrocephala Koidz* (Baizhu), *Hedyotis Diffusae Herba* (Baihuasheshecao), *Curcumae Rhizoma* (ezhu), *Licorice* (Gancao), *Hedysarum multijugum Maxim* (Huangqi), *Solanum nigrum Linn* (Longkui), *Pseudobulbus Cremastrae Seu Pleiones* (Shancigu), *Salviae Chinensis Herba* (Shijianchuan), *Pseudostellariae Radix* (Taizishen), *Tetrapanacis Medulla* (Tongcao) and *Coicis Semen* (Yiyiren).

The information of molecular target filtering was employed to Traditional Chinese Medicines for Systems Pharmacology Database and Analysis Platform (TCMSP, http://lsp.nwsuaf.edu.cn/tcmsp.php) [26].

### Pharmacokinetic prediction

The properties of absorption, distribution, metabolism and excretion (ADME) were considered as important indicators for effectiveness in herbs. According to publications, three ADME-related models, including the evaluation of oral bioavailability (OB), Caco-2 permeability and drug-likeness (DL), are applied to identify the potential bioactive compound of FZKA. Each of property in above was illustrated as following:

OB represents fraction of the oral dose of bioactive compound which reaches systemic circulation in the TCM remedy. The reasonable threshold of OB was set to 33% for further analysis. And the threshold of OB referred to previous studies and used their criterion [27–30]. The indicator of Caco-2 widely applied as standard permeability-screening assay for prediction of the compound’s intestinal absorption and fraction of oral dose absorbed in humans. In this study, the threshold of Caco-2 permeability was set to 0.4 [31]. Drug-likeness evaluation is used in drug design to evaluation whether a compound is chemically suitable for drug, and how drug-like a molecule is with respect to parameters affecting its pharmacodynamic and pharmacokinetic profiles which ultimately impact its ADME properties. In this study, the threshold of DL was set to 0.18 [32].

### Differential expression genes between sensitive and resistance EGFR-TKI lung cancer cells

The differential expression genes (DEGs) between EGFR-TKI sensitive and resistance were calculated from public dataset of Gene Expression Omnibus (GEO). GSE34228 dataset include gefitinib sensitive (*n* = 26) and resistance (*n* = 26) PC9 cell lines. For assay the deregulation gene expression, “limma” package of R software was employed to test the DEGs. mRNAs with log_2_ fold change |log FC| ≥1 (FDR adjusted *P* < 0.01) were considered to be differentially expressed mRNAs.

### Identify core sub-network from compound-target network

For identifying core targets from compounds-targets network, the overlap of DEGs and targets were extracted. There eight overlap targets were identified. We selected eight targets and their neighbors (only compounds) as core sub-network.

### Validation compound-target interaction via docking simulation

Molecular docking simulation was performed to validate those interactions and guide the associated drug discovery in the Glide module of Schrödinger software (Version, Schrödinger, Inc., New York, NY, 2015). The studied compounds were prepared and optimized in the LigPrep module. The crystal structures of the studied protein targets were derived from the protein data bank (PDB) database (http://www.rcsb.org/) and prepared using the Protein Preparation Wizard. The centroid of the co-crystalized inhibitor in the crystal structures of complex was defined as the binding site. The poses of the studied compounds are evaluated by both standard precision and extra precision (XP) docking score and the binding conformation with the highest score was selected for binding mode analysis.

### Validation in cohort with EGFR mutation

The gene expression, samples with EGFR mutation and clinical information of lung adenocarcinoma (LUAD) were downloaded from TCGA database (https://portal.gdc.cancer.gov/). For filtering EGFR mutation samples, we selected the samples which both contain EGFR mutation and complete follow-up information (overall survival). After filtering the samples, there are forty-five samples were included for further analysis. In addition, the patients with relapse free survival (RFS) time information were also selected to validate. There were thirty-seven patients with RFS were filtered to test.

### Risk sore construction

A risk score (RS), linear combination of candidate mRNAs (targets of active compounds) for each LUAD patient with EGFR mutation (*n* = 45), is constructed. The RS was calculated from sum of the expression value of the mRNAs weight multiplied by univariate Cox regression coefficients.

$$ \mathrm{Risk}\ \mathrm{score}\ \left(\mathrm{RS}\right)={\sum}_i{V}_i\times {\beta}_i $$

where *β*_*i*_ represents the Cox regression coefficient of the *i*th variable, and *V*_*i*_represents the value of the ith variable. Where *V*_*i*_ is the log 2-transformed expression value of every mRNA and *β*_*i*_ is the univariate Cox proportional hazards regression coefficient of the *i*th mRNA.

### Survival analysis and receiver operating characteristic curves

The prognostic performance was measured using the area under the curve (AUC) derived from time-dependent receiver operating characteristic (ROC) analysis, and the accuracy of the risk score to predict overall survival (OS) at 3 years was assessed. And risk score to predict RFS at 3 years was also tested. The capacity of the model was evaluated by analysis of area under curve (AUC) of the receiver operating characteristic (ROC) curves. Generally, the value of AUC is between 0.5 and 1, and the larger AUC represents a better performance. The value of AUC is greater than 0.7. It is considered that the model has good capacity in classification. The risk of patient group was classified into two groups (a sensitive and a resistance group) according to median value point of individual patient RS. All statistical analyses were conducted using R Software (Version 3.4.2). Survival curves and ROC curves were generated by the ‘survminer’ [33], ‘survival’, and ‘survivalROC’ packages [34].

### Network visualization and KEGG enrichment

The networks were constructed by Cytoscape software version 3.6.1, which is an open source software for network visualization and analysis [35]. In the network, the compounds and targets are showed by nodes, and the interaction between two nodes is represented by an edge. KEGG analysis were carried out using clusterProfiler package in R (v3.4.2) [36]. KEGG pathways visualization was employed to CyKEGGParser application (Version 1.2.9) in Cytoscape software.

## Results

### Design of workflow

**Fig. 1:**
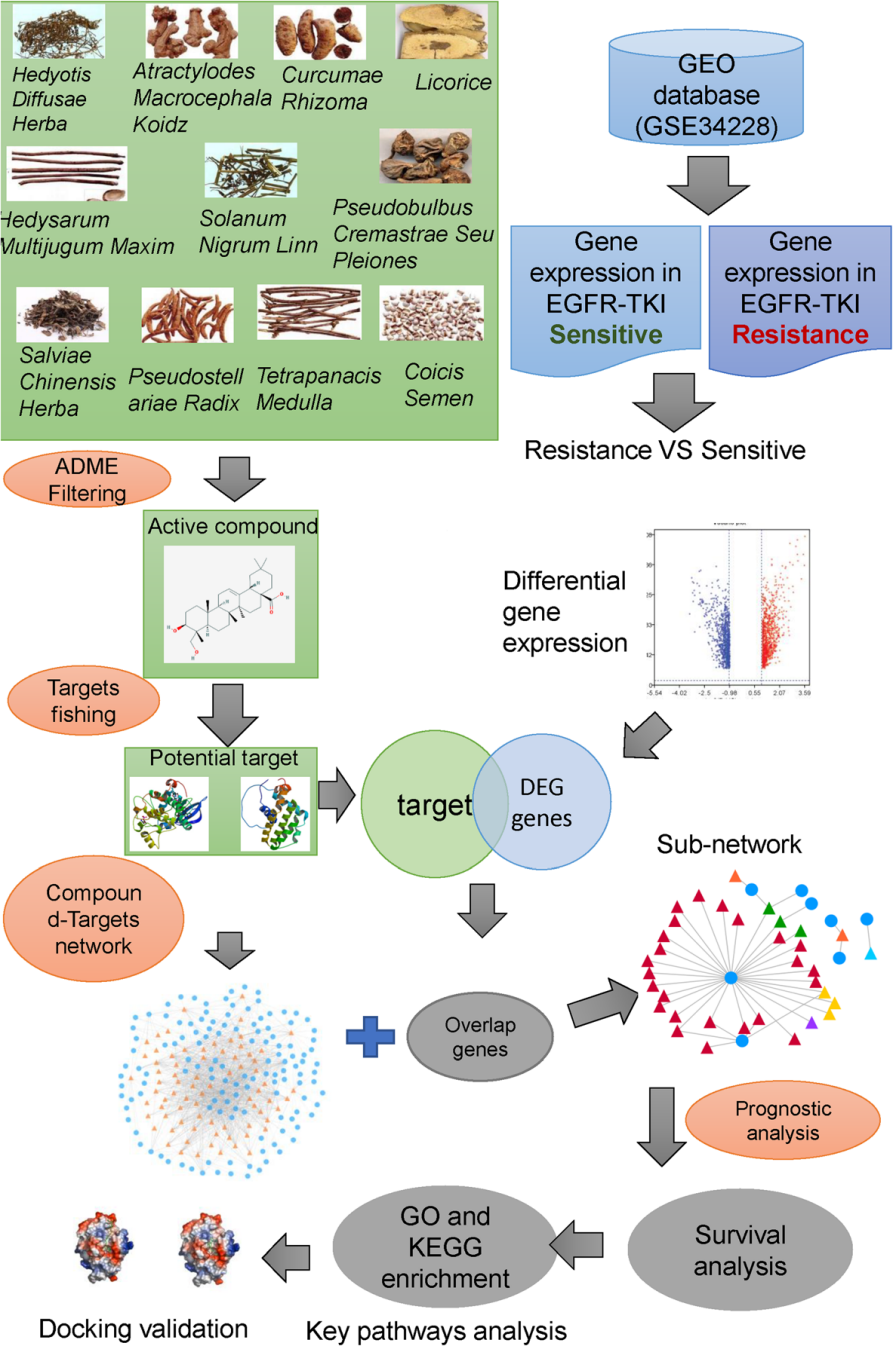
Flowchart of data analysis

For investigating the mechanism of FZKA in EGFR-TKI resistance in molecular level, a hypothesis was proposed, which assumes that targets of bioactive compounds may involve in some pathways that against the EGFR-TKI. In addition, the overlap of targets and DEGs between resistance and sensitive EGFR-TKI NSCLC cell lines would be core targets of herbs. Generally, LUAD patients with sensitive for EGFR-TKI would have better prognosis than resistance. Thus, the prognosis of overlap was validated in LUAD patients with EGFR mutation form TCGA database. For further analysis the pathway of targets, KEGG pathway analysis was used to investigate the active pathway which involved in EGFR-TKI resistance. Finally, molecular docking simulation was employed to validate the interaction of compound and target. The docking simulation can explain the mechanism of interaction how to affect the pathway (Fig. 1).

### Active compounds filtering

**Fig. 2:**
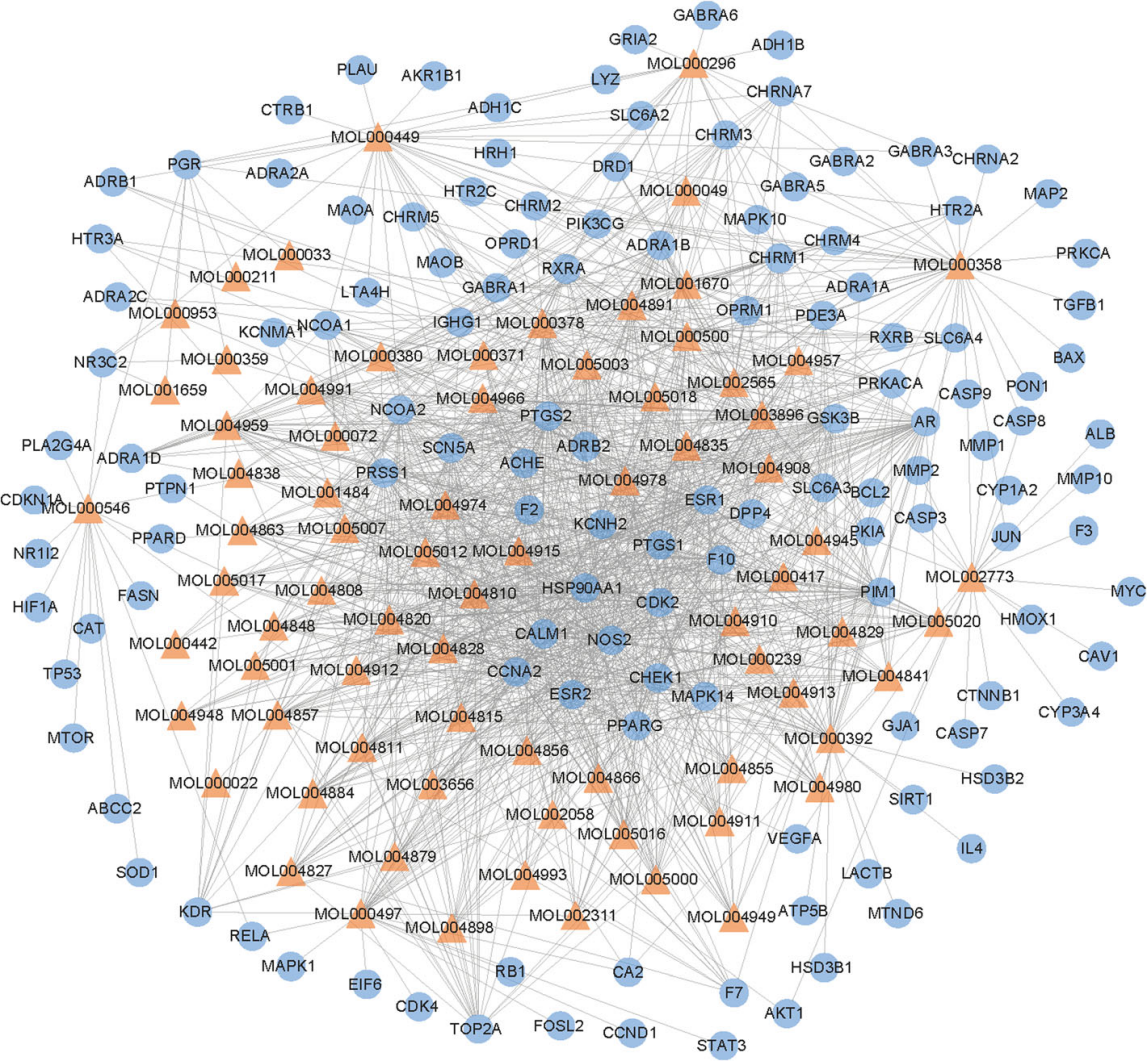
A compound node and a protein node are linked if the protein is targeted by the corresponding compound. Node size is proportional to its degree

After filtering by criterion of ADME in method, a total of 76 compounds were filtered from the eleven herbs of FZKA (Additional file 1). From TCMSP database, the compound-target network was constructed from 76 compounds and 130 targets (Additional file 2). And the network was showed as following (Fig. 2).

The complex network suggested each compound could affect many targets, which can regulate those targets to affect biological process. Although the network showed compound-target interaction, the mechanism in EGFR-TKI resistance LUAD cells was difficult to understand. Thus, the DEGs of sensitive and resistance in LUAD were calculated for further investigation mechanism of herbs.

### Genes associated with Gefitinib resistance

**Fig. 3:**
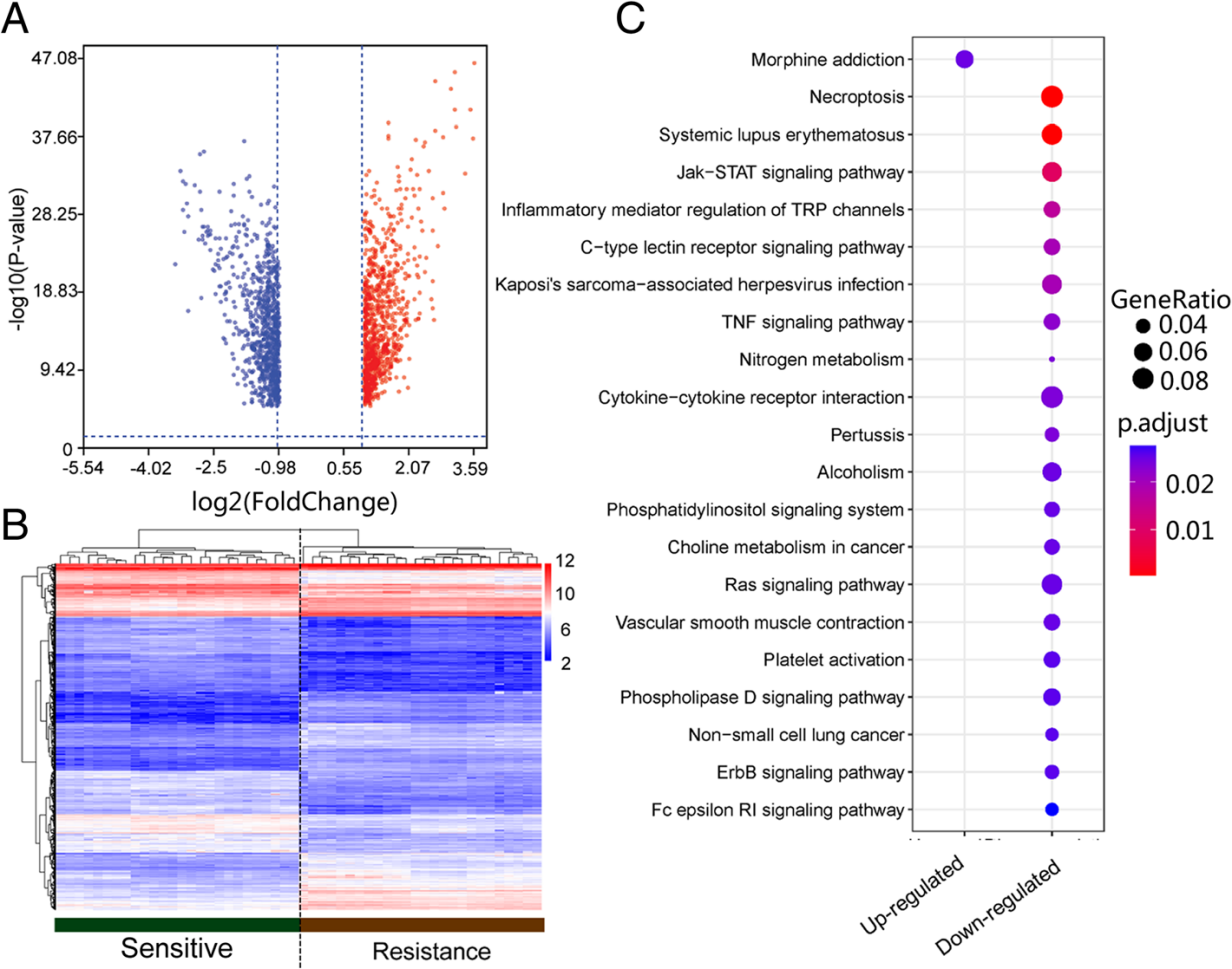
The information of DEGs and pathway of DEGs. **a** Volcano plot represents DEGs. **b** Heatmap of DEGs between sensitive and resistance groups. **c** KEGG enrichment of DEGs

Gefitinib sensitive and resistance cell lines were collected from GSE34228. After filtering by some criterions as methods, there were 449 up-regulation genes and 531 down-regulation genes (Fig. 3a, b). The pathway enrichment of DEGs was employed to KEGG **(**Fig. 3c).

The volcano plot and heatmap showed fold-changes and DEGs in two groups. KEGG enrichment suggested that down-regulated genes involved in more pathways. Of these pathways, some pathways were closely associated with cancer development such as Jak-STAT signaling pathway, TNF signaling pathway, Ras signaling pathway and ErbB signaling pathway. For investigating more accurate targets of FZKA, we intersected the differentially expressed genes and drug targets.

### Core genes identification and network analysis

**Fig. 4:**
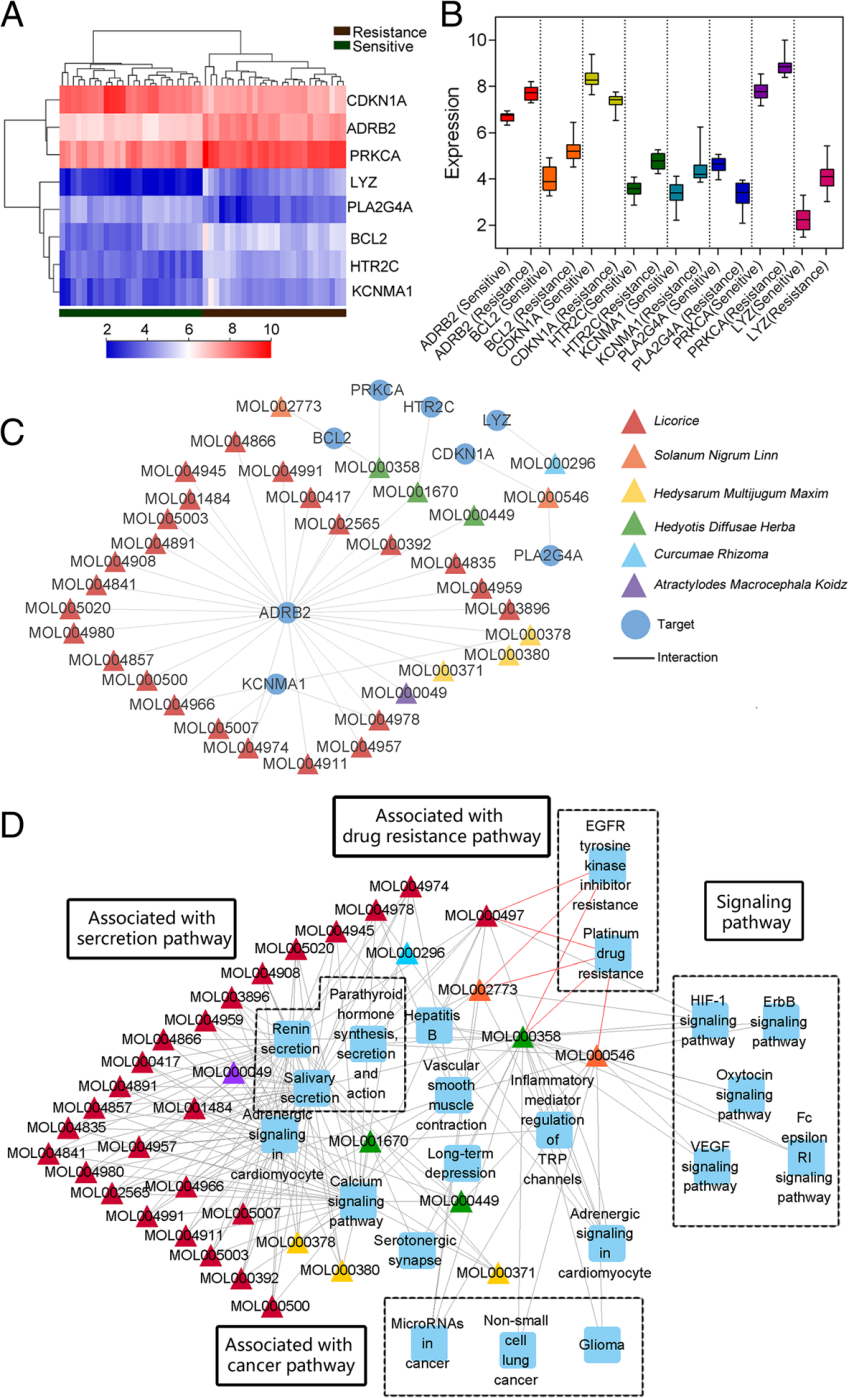
Expression of core genes and subnetwork of core genes. **a** Heatmap of core genes in EGFR-TKI resistance and sensitive groups. **b** boxplot of each core gene between two groups. **c** Subnetwork of compounds and targets. The triangles represent different compounds and different color represent herbs that include the compounds. **d** The compound-pathway interaction network is constructed by compound and the pathway that consisted of core targets. Red lines represent compounds directly related to drug resistance

For identification some core genes in EGFR-TKI resistance of NSCLC, the overlapping genes between targets and DEGs were identified. Eight overlap genes and thirty-five compounds were identified (Tables 1 and 2). The expression of core genes and subnetwork of compound-target network were showed in Fig. 4.

**Table 1:** Information of active compound by ADME filtering

ID	compounds	Structure	OB	Caco.2	DL	Herbs
MOL000049	3β-acetoxyatractylone		54.07	1.13	0.22	Atractylodes Macrocephala Koidz.
MOL000296	hederagenin		36.91	1.32	0.75	Curcumae Rhizoma
MOL000358	beta-sitosterol		36.91	1.32	0.75	Hedyotis Diffusae Herba
MOL000371	3,9-di-O-methylnissolin		53.74	1.18	0.48	*Hedysarum multijugum* Maxim.
MOL000378	7-O-methylisomucronulatol		74.69	1.08	0.3	Hedysarum Multijugum Maxim.
MOL000380	(6aR,11aR)-9,10-dimethoxy-6a,11a-dihydro-6H-benzofurano[3,2-c]chromen-3-ol		64.26	0.93	0.42	Hedysarum Multijugum Maxim.
MOL000392	Formononetin		69.67	0.78	0.21	licorice
MOL000417	Calycosin		47.75	0.52	0.24	licorice
MOL000449	Stigmasterol		43.83	1.44	0.76	Hedyotis Diffusae Herba
MOL000497	licochalcone a		40.79	0.82	0.29	licorice
MOL000500	Vestitol		74.66	0.86	0.21	licorice
MOL000546	Diosgenin		80.88	0.82	0.81	*Solanum nigrum* Linn
MOL001484	Inermine		75.18	0.89	0.54	licorice
MOL001670	2-methoxy-3-methyl-9,10-anthraquinone		37.83	0.73	0.21	Hedyotis Diffusae Herba
MOL002565	Medicarpin		49.22	1	0.34	licorice
MOL002773	beta-carotene		37.18	2.25	0.58	Solanum Nigrum Linn
MOL003896	7-Methoxy-2-methyl isoflavone		42.56	1.16	0.2	licorice
MOL004835	Glypallichalcone		61.6	0.76	0.19	licorice
MOL004841	Licochalcone B		76.76	0.47	0.19	licorice
MOL004857	Gancaonin B		48.79	0.58	0.45	licorice
MOL004866	2-(3,4-dihydroxyphenyl)-5,7-dihydroxy-6- (3-methylbut-2-enyl)chromone		44.15	0.48	0.41	licorice
MOL004891	shinpterocarpin		80.3	1.1	0.73	licorice
MOL004908	Glabridin		53.25	0.97	0.47	licorice
MOL004911	Glabrene		46.27	0.99	0.44	licorice
MOL004945	(2S)-7-hydroxy-2-(4-hydroxyphenyl)-8-(3-methylbut-2-enyl) chroman-4-one		36.57	0.72	0.32	licorice
MOL004957	HMO		38.37	0.79	0.21	licorice
MOL004959	1-Methoxyphaseollidin		69.98	1.01	0.64	licorice
MOL004966	3’-Hydroxy-4’-O-Methylglabridin		43.71	1	0.57	licorice
MOL004974	3′-Methoxyglabridin		46.16	0.94	0.57	licorice
MOL004978	2-[(3R)-8,8-dimethyl-3,4-dihydro-2H-pyrano[6,5-f]chromen-3-yl]-5-methoxyphenol		36.21	1.12	0.52	licorice
MOL004980	Inflacoumarin A		39.71	0.73	0.33	licorice
MOL004991	7-Acetoxy-2-methylisoflavone		38.92	0.74	0.26	licorice
MOL005003	Licoagrocarpin		58.81	1.23	0.58	licorice
MOL005007	Glyasperins M		72.67	0.49	0.59	licorice
MOL005020	dehydroglyasperins C		53.82	0.68	0.37	licorice

**Table 2:** Core targets of Fuzhengkangai

Uniprot accession	Gene names	Protein name	Log (Fold Change)	Adjust *P* value
P07550	*ADRB2*	Beta-2 adrenergic receptor	1.02	1.63e-17
P10415	*BCL2*	Apoptosis regulator Bcl-2	1.20	1.49e-9
P38936	*CDKN1A*	Cyclin-dependent kinase inhibitor 1	−1.01	4.79e-12
P28335	*HTR2C*	5-hydroxytryptamine receptor 2C	1.21	2.58e-16
Q12791	*KCNMA1*	Calcium-activated potassium channel subunit alpha-1	1.01	1.70e-8
P47712	*PLA2G4A*	Cytosolic phospholipase A2	−1.33	3.70e-8
P17252	*PRKCA*	Protein kinase C alpha type	1.06	3.69e-13
P61626	*LYZ*	Lysozyme C	1.91	4.52e-17

The results showed that 35 compounds acted on 20 pathways, which showed average degree of 6.32. In the compound-pathway network, the compounds mainly involved in pathways such as Salivary secretion (degree = 33), Renin secretion (degree = 32) and Calcium signaling pathway (degree = 31) (Additional file 3).

As shown in Fig. 4d, we clustered the pathways into four modules which were secretion pathways, drug resistance pathways, cancer pathways and signaling pathways. The compounds of FZKA have multiple target effects and involved in multiple pathways that may be related to drug resistance. Of these compounds, MOL000497 (licochalcone a), MOL002773 (beta-carotene), MOL000358 (beta-sitosterol) and MOL000546 (diosgenin) directly involved in drug resistance pathways (EGFR-TKI resistance and platinum drug resistance).

### Core genes validation in independent dataset

**Fig. 5:**
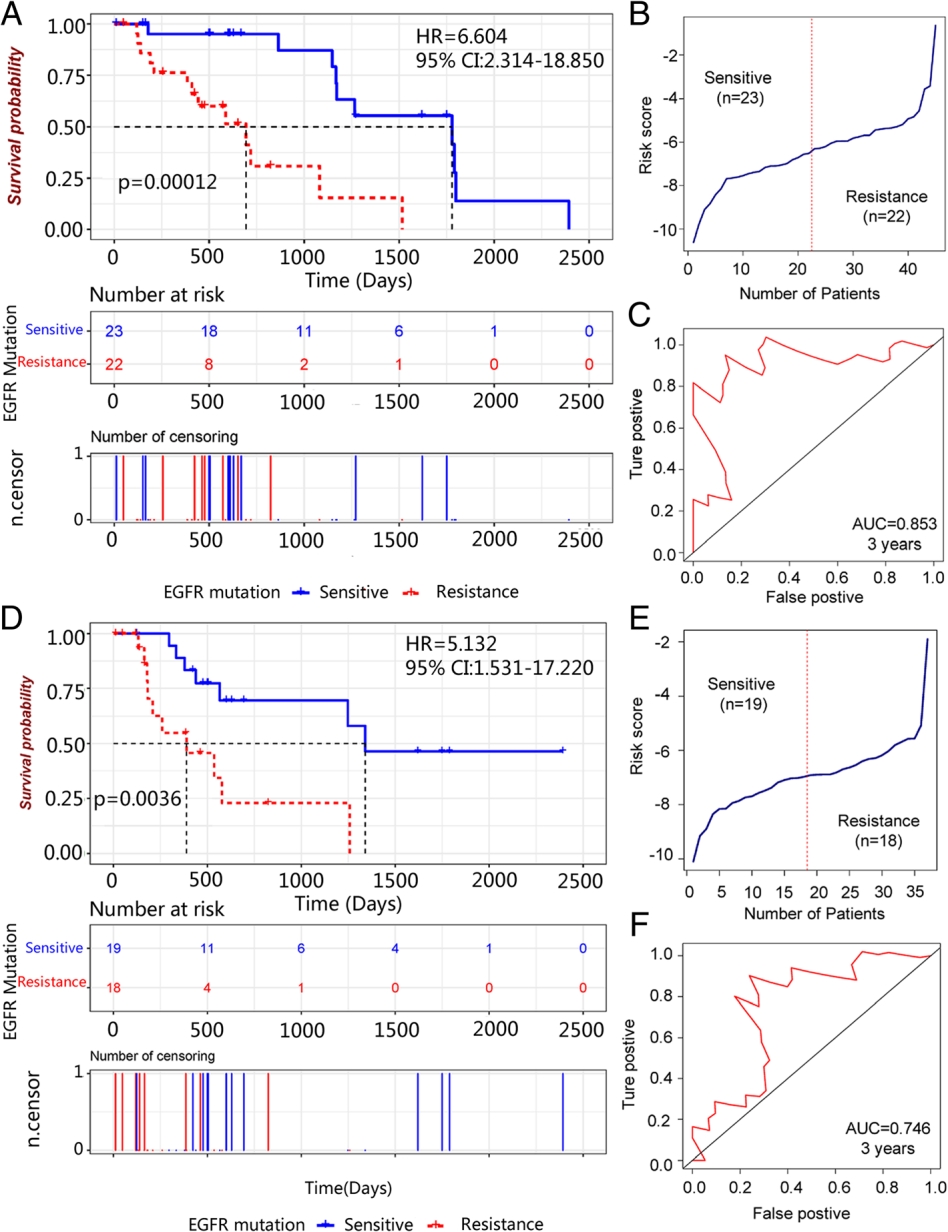
Core genes prognostic validation in LUAD with EGFR-mutation cohort. **a** Kaplan-Meier survival curve of sensitive and resistance groups for overall survival. **b** RS distribution in all mutation patients. **c** AUC of ROC for predicting RS of OS (AUC = 0.853). **d** Kaplan-Meier survival curve of sensitive and resistance groups for disease free survival. **e** RS distribution in all mutation patients with disease free survival information. **f** AUC of ROC for predicting RS of DFS (AUC = 0.746)

The core genes were searched from EGFR-mutation LUAD cohort in TCGA. The clinical factors and baseline information of these patients were listed in Table 3. And RS of overall survival is calculated from linear combination of gene expression and coefficient. The median value of RS is considered as threshold to classify patients into two groups and RS was significantly associated with LUAD patient survival (HR = 6.604, 95% CI: 2.314–18.850). Generally, sensitive group would have longer survival time than resistance. The median value of RS showed that survival of sensitive group was significantly better than resistance group (*p* = 0.0012) (Fig. 5a). The RS distribution in patients was showed in Fig. 5b. And AUC of RS showed that the core genes model performed (AUC = 0.853) well prediction capacity (Fig. 5c). Additionally, RS of RFS was also validated in TCGA database (Fig. 5d). The result of RS in RFS was similar to overall survival (HR = 5.132, 95% CI: 1.531–17.220). And the log-rank test showed that these genes could significantly classify patients into two groups (*p* = 0.0036). AUC of RS of RFS also showed well prediction capacity in 3 years (AUC = 0.746).

**Table 3:** Baseline information of NSCLC patients with EGFR mutation

Clinical factors	Patients (%)	Death	Log-rank test, p
Age			
> =65	24 (53.3%)	14	0.70
< 65	21 (46.7%)	9	
Gender			
Male	13 (28.9%)	7	0.6
Female	32 (71.1%)	16	
Race			
Asian	2 (4.4%)	1	0.6
Black or African American	3 (6.7%)	1	
Not reported	4 (8.9%)	1	
White	36 (80.0%)	20	
Cancer Status			
Tumor free	20 (44.4%)	6	0.01*
With tumor	15 (33.3%)	12	
Unknow	10 (22.2)	5	
Stage			
Stage I	22 (48.9%)	9	0.6
Stage II	9 (20.0%)	5	
Stage III	11 (24.4%)	8	
Stage IV	2 (4.4%)	1	
Not reported	1 (2.2%)	0

### KEGG pathway enrichment

**Fig. 6:**
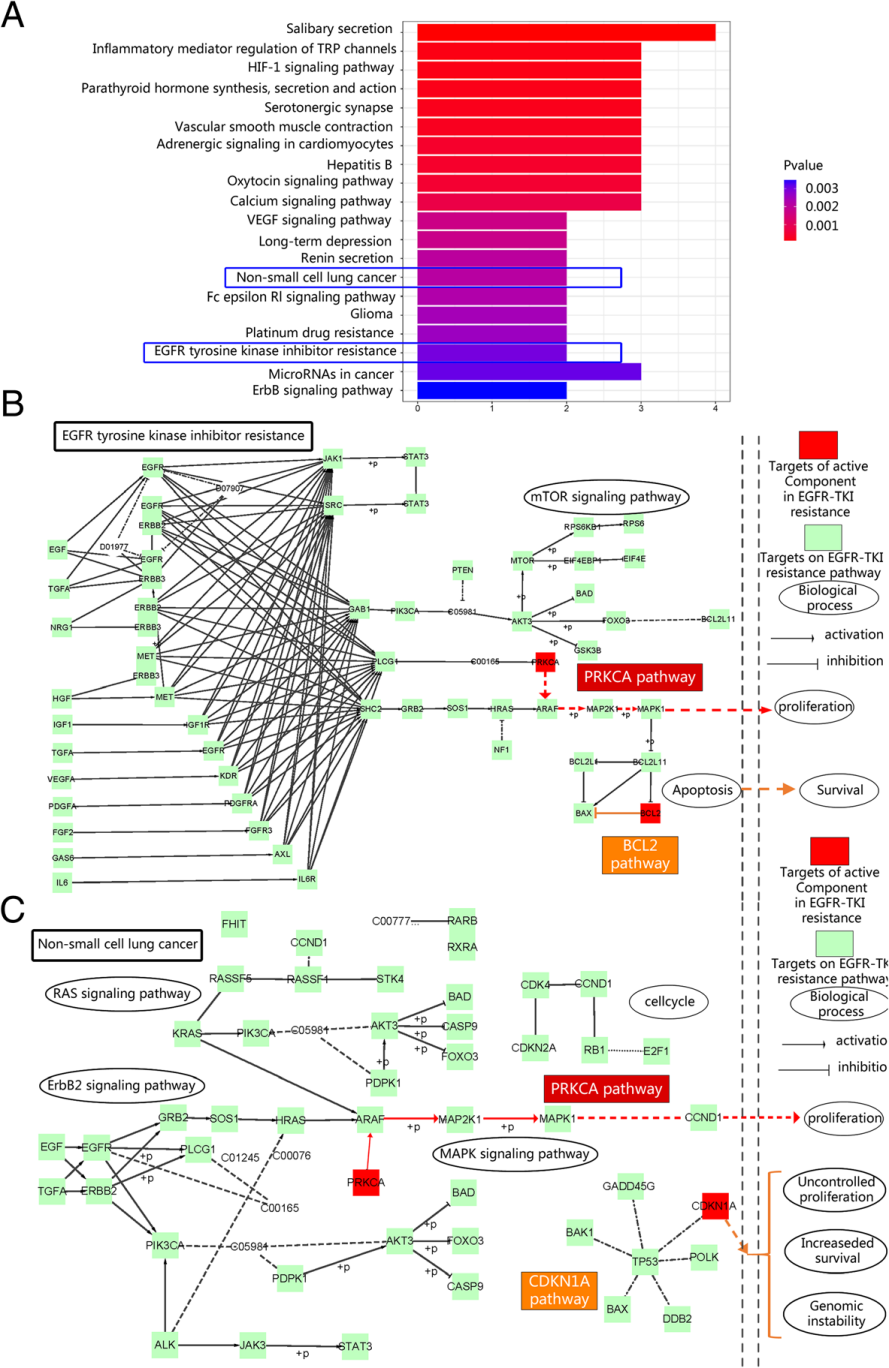
KEGG enrichment of core genes. **a** KEGG pathway enrichment of core genes. **b**. EGFR tyrosine kinase inhibitor resistance pathway. **c** Non-small cell lung cancer pathway

The results of pathway enrichment will be able to show how these drugs act on the pathway, thereby alleviating cell resistance to drugs (Fig. 6). Through the result of KEGG pathway enrichment showed that two pathways (hsa05223: Non-small cell lung cancer and hsa01521: EGFR tyrosine kinase inhibitor resistance) were directly associated with EGFR-TKI resistance.

The EGFR tyrosine kinase inhibitor resistance pathway showed that there were many pathways can lead to drug resistance. In this study, the pathway enrichment indicated that FZKA acted on PRKCA and BCL2 pathway to affect drug resistance. In addition, in Non-small cell lung cancer, FZKA also act on PRKCA and CDKN1A (p21).

### Molecular docking assay

The mechanism of FZKA was reflected by interaction of compound and target. Thus, molecular docking simulation is used to analyze interaction between them. Three targets and four compounds which involve in the EGFR-TKI resistance were listed in Table 4.

**Table 4:** The genes involved in two pathways for molecular docking

Molecular ID	Molecular names	Protein name	Uniprot ID	Gene symbol
MOL000358	beta-sitosterol	Protein kinase C alpha type	P17252	*PRKCA*
MOL000358	beta-sitosterol	Apoptosis regulator Bcl-2	P10415	*BCL2*
MOL000546	diosgenin	Cyclin-dependent kinase inhibitor1	P38936	*CDKN1A*
MOL002773	beta-carotene	Apoptosis regulator Bcl-2	P10415	*BCL2*
MOL000491	licochalcone a	Apoptosis regulator Bcl-2	P10415	*BCL2*

**Fig. 7:**
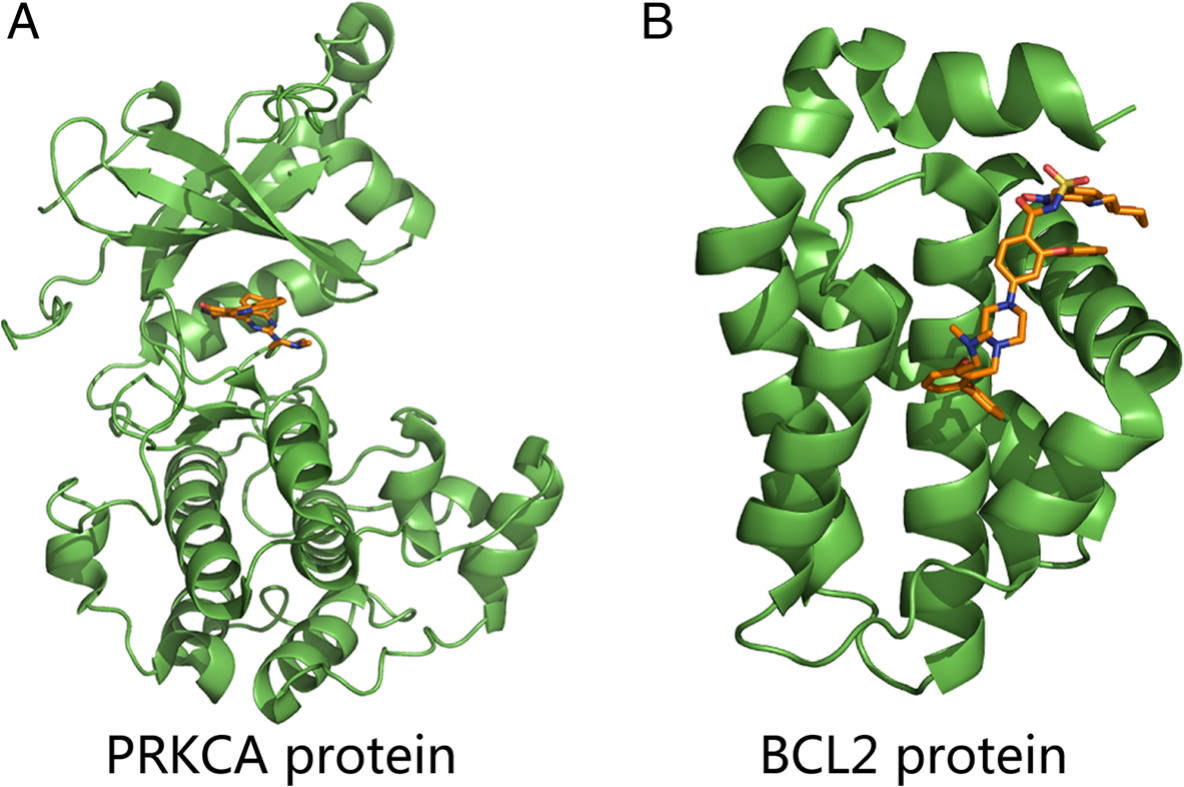
The binding sites of PRKCA and BCL2. **a** Three-dimension structure of PRKCA. **b** Three-dimension structures of BCL2. The proteins are shown in green cartoon and the co-crystalized inhibitors are shown in orange sticks

The versatile functions of CDKN1A (p21) are not fully understood and the associated pathways and mechanism need to be further elucidated (a. Less understood issues: p21Cip1 in mitosis and its therapeutic potential; b. Ironing out the role of the cyclin-dependent kinase inhibitor, p21 in cancer: Novel iron chelating agents to target p21 expression and activity). The structure and the interacting information of active pocket of CDKN1A (p21) are still lacking. Thus, we mainly concentrated our docking analysis on PRKCA and BCL2. The 3D structure of PRKCA and BCL2 are derived from the PDB database and used for docking analysis (Fig. 7).

In simulation processing, CDKN1A (p21) does not have full-length crystal structure and active pocket information. Thus, we searched 3D structure of PRKCA and BCL2 for docking analysis.

**Fig. 8:**
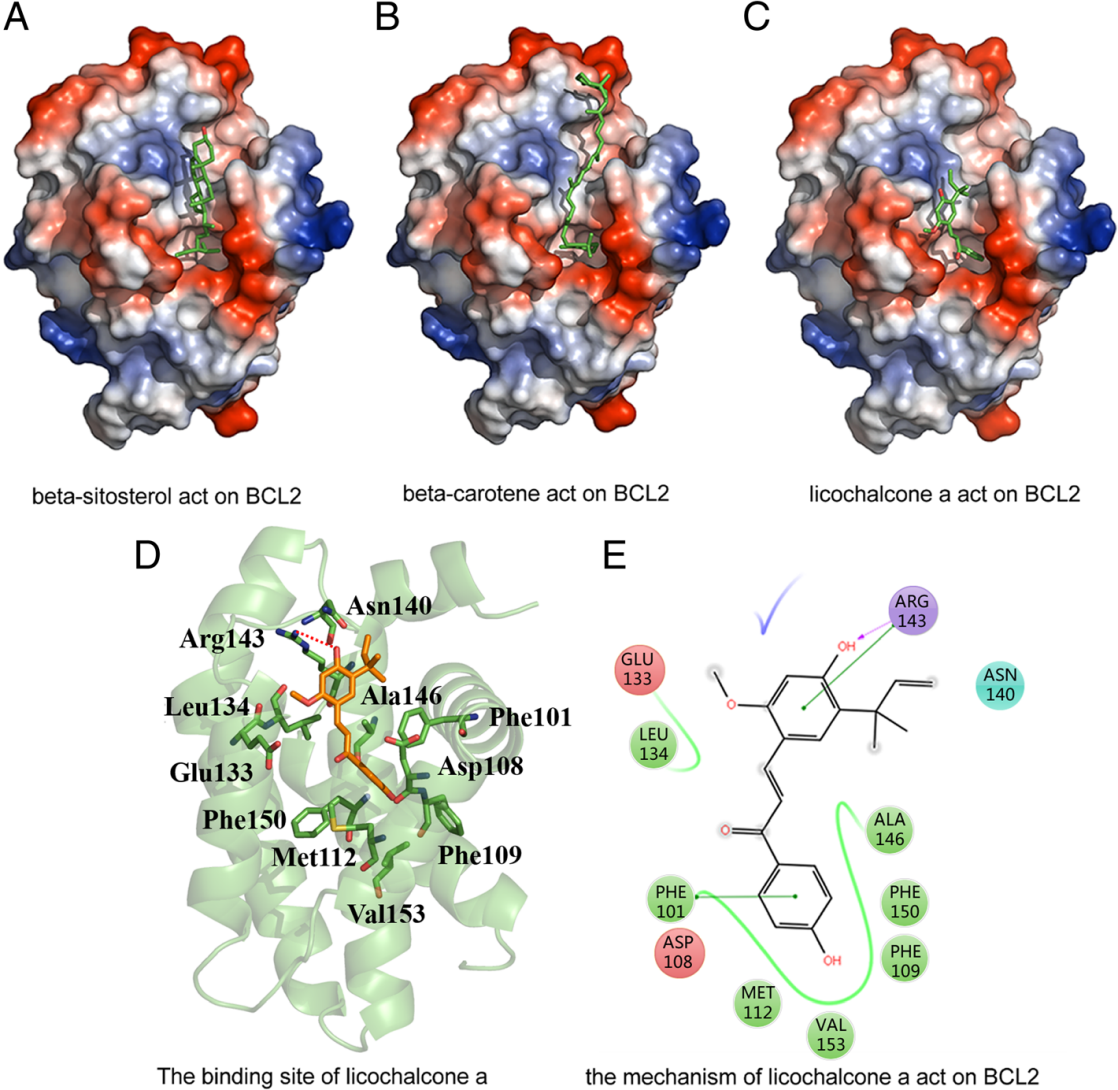
The interactive modes of beta-sitosterol, beta-carotene, licochalcone a with BCL2. **a** beta-sitosterol binding on the pocket of BCL2. **b** beta-carotene binding on the pocket of BCL2. **c** licochalcone a binding on the pocket of BCL2. **d** the three-dimensional representation of the binding mode of licochalcone a with BCL2. The residues in the binding site are shown in green sticks and licochalcone a is shown in orange sticks. **e** the electrical characteristics of the surrounding residues of licochalcone a and the interaction between licochalcone a and BCL2. The green, cyan, red, blue circles represent hydrophobic, polar, negative-charged, positive-charged residues

PRKCA protein has a common characteristic of kinases and a small-molecule ligand is bound to ATP’s competitive pockets [37]. During the molecular docking simulation, we docked beta-sitosterol into the binding pocket of PRKCA protein, however, it returned no binding poses of beta-sitosterol, which indicates that beta-sitosterol doesn’t have the ability to bind to the active site of PRKCA protein. The binding pocket of BCL2 shows that it has the characteristics of protein-protein interaction [38]. The results of docking simulation for BCL2 suggested that all the three small-molecule ligands could be docked to the binding site of BCL2 as shown in Fig. 8a, b and c. LCA tends to have the best affinity among three compounds according to the docking scores (Table 5) and the detailed binding mode of LCA was analyzed. As shown in Fig. 8d, e, LCA was buried in a hydrophobic pocket formed by Phe101, Asp108, Phe109, Met112, Glu133, Leu134, Asn140, Arg143, Ala146, Phe150, Val153. Among these residues, the hydroxy of LCA had a hydrogen bonding with Arg143, while the benzene rings of LCA formed π-π stacking and π-cation interaction with Phe101 and Arg143. The results of the molecular docking simulation above showed that BCL2 tended to be the potential target involved in EGFR-TKI resistance and non-small cell lung cancer pathways and LCA could be an active compound to decrease the EGFR-TKI resistance.

**Table 5:** SP- and XP-Docking score of three compounds

Molecular ID	Compound name	SP-docking	XP-docking
MOL000358	beta-sitosterol	−4.446	−4.542
MOL000546	beta-carotene	−3.845	−4.925
MOL000497	licochalcone a	−5.339	−5.312

## Discussion

Recently, with the growing research on CHM by network, a new “multi-target, multi-drug” model was considered as more effective strategy for understanding drug action and treatment complex disease [39]. Although a CHM formulation of FZKA was reported for treatment NSCLC patients with EGFR-TKI resistance, the mechanism o formulations have not been illustrated. In this work, we employed complex network analysis, bioinformatics and computer simulation methods for investigating the mechanism of drug action. The results suggested that one of the main mechanisms may be by inhibiting BCL2 and PRKCA pathway which were EGFR-TKI resistance pathways for overcoming EGFR-TKI resistance.

Generally, many studies considered that hub targets or hub pathways interacted with compounds in the network as an important point in drug action. In this study, ADRB2 is a hub node in the subnetwork. The subnetwork showed that there were many molecules interacting with ADRB2 (Fig. 4c). Beta-2 adrenergic receptor (ADRB2), coded by an intronless gene on chromosome 5q31–32, mediate the catecholamine-induced activation of adenylate cyclase through the action of G proteins [40]. Generally, ADRB2 was reported that it significantly associated development of cancer and it is considered that sympathetic neurotransmitters can act as ligands and activate ADRB2 expressed on the surface of tumor cells to promote tumor growth [41]. In addition, ADRB2 was also found that it associated with risk of asthma and LUAD [42–44]. At present, the relationship between ADRB2 and lung cancer is mainly related to the activation of mitotic pathways [44]. And some studies figure out activity ADRB2 can active EGFR signaling pathway for tumor growth [45, 46]. Although ADRB2 was not directly involved in EGFR-TKI resistance pathway in this study, the role of ADRB2 was very important in EGFR-TKI resistance due to involve in cell proliferation and EGFR signaling pathway.

The proteins involved in important biological pathways was considered as core proteins. And the compounds interacted with core proteins may be key component in herbs. There eight genes were searched from overlap of targeted proteins and DEG from sensitive and resistance PC9 cell lines. And eight genes can predict prognosis of LUAD patients with EGFR mutation. And RS of these genes can significantly classify the patients into sensitive and resistance groups (Fig. 6).

Of these genes, BCL2, PRKCA (PKC) and CDKN1A (p21) were directly associated with EGFR-TKI resistance pathway (hsa01521) and NSCLC pathway (hsa05223). And five compounds act on these proteins (Table 5). BCL2 is a noted protein in regulation apoptosis of cancer. Previous study reported that overexpression BCL2 can inhibit apoptosis in cancer cells [47]. And BCL2 was also reported that it involved in the mediation of chemotherapy resistance in NSCLC [48]. In this study, EGFR-TKI resistance cell lines have high expression of BCL2 (Fig. 4b). In addition, the results showed that three compounds acted with BCL2. And licochalcone a (LCA) showed the best score in three compounds. According to previous study, LCA can inhibit BCL2 for inducing autophagy and promoting apoptosis in cancer cells [49]. Another study reported that LCA induced autophagy effect in NSCLC cells [50]. Although these two studies reported that LCA can induce apoptosis and autophagy by experiment, the mechanism of molecular level has not been revealed. In our study, the simulation results showed that LCA act on active package of BCL2 protein. The binding of small molecules to BCL2 can influence the binding of BCL to downstream ligands. Therefore, the binding of three small molecules and BCL2 may play vital role in regulation apoptosis of LUAD with EGFR-TKI resistance.

Other two proteins (PRKCA and CDKN1A) were also analyzed in study. But 3D structure of CDKN1A(p21) has not been resolved yet. So, the mechanism of CDKN1A fail to analyze. Additionally, in analysis of PRKCA, the binding of beta-sitosterol and PRKCA was very different from common PRKCA inhibitor. So, the molecular simulation software doesn’t get the result from PRKCA. Although PRKCA and CDKN1A have not been validation by molecular simulation, the results also indicated that FZKA could overcome EGFR-TKI resistance through affecting eight core targets. Of these targets, ADRB2, BCL2, PRKCA and CDKN1A were reported by previous publications. Other genes have not been reported to associated with EGFR-TKI in LUAD.

Above all, from our analysis, the compounds from *Hedyotis Diffusae Herba*, *licorice*, *Hedysarum multijugum Maxim*, *Solanum nigrum Linn*, *Curcumae Rhizoma* and *Atractylodes Macrocephala Koidz* play major role in overcoming EGFR-TKI resistance in LUAD. And BCL2 and PKC pathways may be main targets of FZKA. And these two targets as drug targets for overcoming EGFR-TKI resistance were also reported by previous publication. Other targets such as LYZ, HTR2C, KCNMA1 and PLA2G4A were not enriched in pathway that related with EGFR-TKI resistance. However, these targets may be potential targets of drug resistance. Further experiments are still needed confirm this conclusion.

## Conclusion

In clinical practice, it has been found that FZKA has the effect of overcoming the drug resistance of EGFR mutations positive, but the molecular mechanism is unclear. This study revealed that compounds from FZKA directly acted on targets which involved in EGFR-TKI resistance. That interaction indicated that FZKA can overcome drug resistance through inhibiting BLC2 and PRKCA pathways.

## Additional files

Additional file 1: The details information of 76 compounds that were filtered by ADME from the eleven herbs of FZKA. (XLSX 16 kb)

Additional file 2: The compound-target network was consisted by 76 compounds and 130 targets. (XLSX 14 kb)

Additional file 3: The information of compound-pathway network obtained with network analysis. (XLSX 16 kb)

## Abbreviations

ADME: Absorption, distribution, metabolism and excretion
AUC: Area Under roc Curve
CHM: Chinese herbs medicine
DEGs: Differential gene expressions
DL: Drug-likeness
EGFR: Epidermal growth factor receptor
EGFR-TKI: Epidermal growth factor receptor tyrosine kinase inhibitors
FZKA: Fuzhengkangai
KEGG: Kyoto Encyclopedia of Genes and Genomes
LCA: Licochalcone a
LUAD: Lung adenocarcinoma
NSCLC: Non-small cell lung cancer
OB: Oral bioavailability
ROC: Receiver Operating Characteristic
RS: Risk score
